# Multilabel convolution neural network for facial expression recognition and ordinal intensity estimation

**DOI:** 10.7717/peerj-cs.736

**Published:** 2021-11-29

**Authors:** Olufisayo Ekundayo, Serestina Viriri

**Affiliations:** Computer Science Discipline, University of KwaZulu-Natal, Durban, South Africa

**Keywords:** Binary cross-entropy, Facial expression recognition, Island loss, Multilabel, Ordinal intensity estimation

## Abstract

Facial Expression Recognition (FER) has gained considerable attention in affective computing due to its vast area of applications. Diverse approaches and methods have been considered for a robust FER in the field, but only a few works considered the intensity of emotion embedded in the expression. Even the available studies on expression intensity estimation successfully assigned a nominal/regression value or classified emotion in a range of intervals. Most of the available works on facial expression intensity estimation successfully present only the emotion intensity estimation. At the same time, others proposed methods that predict emotion and its intensity in different channels. These multiclass approaches and extensions do not conform to man heuristic manner of recognising emotion and its intensity estimation. This work presents a Multilabel Convolution Neural Network (ML-CNN)-based model, which could simultaneously recognise emotion and provide ordinal metrics as the intensity estimation of the emotion. The proposed ML-CNN is enhanced with the aggregation of Binary Cross-Entropy (BCE) loss and Island Loss (IL) functions to minimise intraclass and interclass variations. Also, ML-CNN model is pre-trained with Visual Geometric Group (VGG-16) to control overfitting. In the experiments conducted on Binghampton University 3D Facial Expression (BU-3DFE) and Cohn Kanade extension (CK+) datasets, we evaluate ML-CNN’s performance based on accuracy and loss. We also carried out a comparative study of our model with some popularly used multilabel algorithms using standard multilabel metrics. ML-CNN model simultaneously predicts emotion and intensity estimation using ordinal metrics. The model also shows appreciable and superior performance over four standard multilabel algorithms: Chain Classifier (CC), distinct Random K label set (RAKEL), Multilabel K Nearest Neighbour (MLKNN) and Multilabel ARAM (MLARAM).

## Introduction

Recognising human affective state from a facial image is one of the most relevant challenges in Computer Vision (CV) and Human-Computer Interaction (HCI). This aspect of Computer Vision has gained much attention; several methods and approaches have been proposed in the literature. Early methods resolved that FER is a multiclass problem and thus proposed multiclass based classifiers or adapted binary classifier to multiclass problems as appropriate methods for FER classification. For instance, Ekman & Friesen (1971) categorised facial expression into six basic emotion classes: Anger, Disgust, Fear, Happy, Sadness and Surprise. This classification automatically restricted FER into a multiclass task and buried much information that could help achieve robustness and better accuracy. The concept of arousal and valence model reveals more information content of FER. While arousal considers the expression intensity, valence captures the pleasantness and the unpleasantness of the expression (Mollahosseini, Hasani & Mahoor, 2019; Yang & Sun, 2017).

The expression intensity can be classified as one of the main attributes of emotion in facial expression. Plutchik (2001) ascertained that expression is a result of combination of basic emotions in the face. Yannakakis, Cowie & Busso (2017) reiterated that in real life, the display of pure emotions is rare and described emotion as a relative notion that should not be classified in terms of absolute values in the standard classification algorithms. Expression recognition and intensity estimation is a common task executed by human beings. Human beings find it easy, convenient, and comfortable to predict the emotional state concurrently and the accompanying intensity (using ordinal metrics) of a person from expression image. This intrinsic ability in human has not been adequately modeled in FER system. The classification of facial expression into basic emotion states has been considered severally in diverse ways in the literature, yet the approach could not account for the intensity of the recognised emotion. Likewise, few studies on emotion recognition and intensity estimation from face image succeeded in assigning numeric values as the estimated intensity. This attempt is far from the perception of man towards emotion intensity estimation. Man has a hierarchical structure perception about emotion and therefore estimate it using referenced base value, which allows its semantics preservation. To the best of our knowledge, none of the works on facial expression recognition and intensity estimation considered static FER dataset, and the environments explored in the study are sequence and dynamic environments. The notion that sequence and dynamic data contain more information of expression intensity and lack of hierarchical annotated static dataset may be the cause. Our findings show that the only static dataset in the field with ordinal annotation is BU-3DFE.

**Figure 1:**
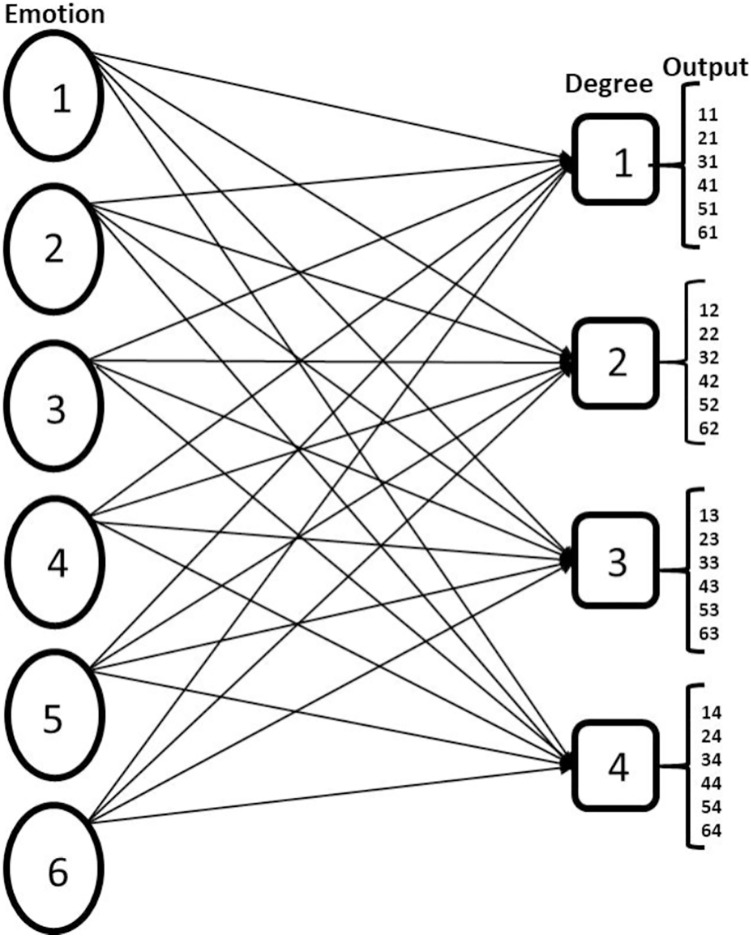
Showing multilabel problem formulation of FER. The nodes under emotion represent the six basic emotion classes Anger, Disgust, Fear, Happy, Sad, Surprise, and the nodes under the degree represent the ordinal estimation of emotion intensity Low, Normal, High, Very High and the output is the possible result of the multilabel CNN classification.

In this study, FER is considered a multilabel problem because an instance of a facial expression image contains information about emotion displays and the corresponding intensity. The six possible emotion states include Anger, Disgust, Fear, Happy, Sadness and Surprise. The ordinal metrics for estimating the category of emotion intensity are: low, normal, high and v_high (very high). The first phase of the FER multilabel approach is data organisation. We organise the data such that each emotional state is associated with the corresponding intensity; this is pictorially represented in Fig. 1. We implement a problem transformation technique using binary relevance (BR). The CNN network with sigmoid function in the output layer serves as the binary classifier. Because of our dataset population, we use the pre-trained network (VGG-16) to avoid model overfitting, which was a challenge in Ekundayo & Viriris (2020). To reduce intraclass variation and increase interclass variation an aggregation loss (combination of island loss and BCE loss) is proposed which is another additional feature to our work in (Ekundayo & Viriris, 2020). The contributions of this work include:

- Multilabel model of facial expression recognition and intensity estimation. With this model, both the emotion features and the hierarchical structure embedded in them are learned concurrently.
- Ordinal metrics are used for the emotion intensity estimation, enabling the model to present the intensity estimation in a similar way like human beings.
- Use of Binary relevance multilabel transformation technique and CNN classifier, CNN is used as a binary classifier by implementing sigmoid function at the network’s output layer. This ensures that the prediction probability of any class is independent of the other classes. Classifier sensitivity to intraclass and interclass variation is enhanced with the aggregation of island loss and BCE loss.

The proposed ML-CNN facial expression recognition model is capable of predicting the emotion and the corresponding ordinal intensity estimation concurrently from facial expression images. The simultaneous prediction of emotion and its intensity is a vital information in the application of FER; especially in psychiatry and schizophrenia (Behere, 2015; Seo et al., 2020) and also for pain (Chen, Ansari & Wilkie, 2012; Roy et al., 2016) and depression analysis (Guo et al., 2021). Application of FER intensity estimation in real- world could mitigate the challenges of recognising emotion in schizophrenia patients, also since pain and depression are categorised as compound emotions (Du & Martinez, 2015) FER intensity estimation could appropriately state the degree to which they are expressed. Quantifying emotion with ordinal metrics makes ML-CNN to be similar to human prediction of emotion, which agrees with adaptation level theory account of Russell on emotion (Russell & Lanius, 1984), and ordinal nature of emotion as presented in Yannakakis, Cowie & Busso (2017).

This work is organised as follows: Section “Related Works” discusses some studies related to both facial expression recognition and intensity estimation, the discussion also covers some of CNN network optimisation techniques. Section “Multilabel Convolution Neural Network Model (ML-CNN) Description” presents the ML-CNN model description; starting from problem formulation to describing the CNN network and the enhance loss functions employed. Section “Experiment” contains details of the experiments, which involve: the preprocessing of the data, and the experiment procedure details, and brief introduction of the databases. In Section “Experimental Results and Discussion”, we provide a logical presentation of the experiments’ result and relevant discussion of the experiments’ outcomes. Section “Conclusions” is the conclusion of the work.

## Related works

In quest of a robust FER system, several studies have been conducted using traditionally handcrafted methods (Li & Deng, 2020; Turan & Lam, 2018), conventional machine learning techniques (Ekundayo & Viriri, 2019) and the state-of-the-art deep learning methods (Liu et al., 2017). The named techniques have been thoroughly considered under the supervised and unsupervised approaches in either a static or dynamic environment. Most of these approaches only succeeded in classifying an expression image into six or seven emotion classes.

Deep learning methods continue to evolve in diverse ways to achieve an optimal result in FER classification, and this is evident in EMOTIW2015, and EMOTIW2016 competition (Fan et al., 2016; Kahou et al., 2015). This section will concentrate more on the deep learning approach to facial expression recognition and intensity estimation, and some optimisation techniques adapted to CNN performance improvement.

FER classification is further extended to expression intensity estimation; few works on emotion intensity estimation are available in the literature; many works concentrate more on action unit intensity estimation. For example; Gudi et al. (2015) proposed a single CNN network for simultaneous estimation of Action Unit (AU) activation and intensity estimation. They claimed that activating the specific neuron of the output layer could result into a binary and continuous classification of AUs and corresponding intensity. Likewise, Batista et al. (2017) proposed AUMP Network (AUMP-NET), this network is a single network with multi-output regression capacity to learn AUs relationship and their respective intensity. The network is capable of learning the available AU and its corresponding intensity, simultaneously. Also, the network could learn to pose feature variations using multitask loss. These methods only determined the occurrence of AUs; the intensity is computed by regression means. The intensity of the AUs is not modelled in the training of the network. Similar studies on AU detection and intensity estimation could be found in Zhao et al. (2016) and Zhou, Pi & Shi (2017).

The few works on emotion recognition and intensity estimation are categorised in Kamarol et al. (2017) as: the distance-based (Verma et al., 2005), the cluster-based (Quan, Qian & Ren, 2014), the graphical-based (Valstar & Pantic, 2012) and the regression-based (Nomiya, Sakaue & Hochin, 2016) methods. As stated earlier, our focus is on recent deep learning approaches to emotion recognition and intensity estimation. Aamir et al. (2020) proposed a multilevel convolution neural network for expression classification and intensity estimation. The proposed deep network has two net phases: the expression-network phase, which handles the classification of facial expression image into the basic classes of emotion, and the intensity-network phase that takes the output of expression-network, which is one of the basic emotion and focus on the determination of the degree at which the recognised emotion is expressed. Summary of the existing method are presented in Table 1.

**Table 1:** Summary of various models for emotion and intensity recognition.

Method	Model	DB & performance	Limitation
Verma et al. (2005)	Distance based	Primary source: NA	Only few emotions are considered, method not generalise, emotion intensity before emotion recognition, computationally expensive.
Lee & Xu (2003)	Optical flow tracking algorithm (Distance)	Real-time data	Need for each subject to be trained differently, not generalise, predicting intensity before emotion
Kim & Pavlovic (2010)	HCORF (Prob)	CMU	Intrinsic topology of FER data is linearly model.
Quan, Qian & Ren (2014)	K-Means (Cluster)	CK+	Predict intensity before emotion, intensity estimation based on graphical difference is not logical
Chang, Chen & Hung (2013)	Scatering transform + SVM (Cluster)	CK+	Emotion recognition task is omitted.
Zhao et al. (2016)	SVOR (Regression)	Pain	Correlations between emotion classes are not modelled.
Rudovic, Pavlovic & Pantic (2012)	LSM-CORF (Prob)	BU-4DFE, CK+	Latent states are not considered in the modeling of sequences across and within the classes
Walecki et al. (2015)	VSL-CRF (Prob)	CK+ AFEW	Result of emotion intensity is not accounted for.
Kamarol et al. (2017)	weighted vote	CK+	Emotion and emotion intensity not concurrently predicted.
Proposed model	ML-CNN (Multi-Label)	BU-3DFE	Assume temporal information among sequence data as ordinal metrics.

**Note:**

NA: Not Applicable, MAE: Mean Absolute Error, PCC: Pearson Correlation Coefficient, ICC: Intraclass Correlation, MAL: MeanAbsolute Loss, HL: Hamming Loss, RL: Ranking Loss; AP: Average Precision, CE: Coverage Error.

Xu et al. (2020) proposed a multitasking learning system using a cascaded CNN, and the objectives tend towards incorporating students attentiveness and students emotion recognition and intensity estimation into an intelligent class system. The first module of the cascaded network handled the preprocessing stages that involve face detection, face alignment and head pose estimation through which attentiveness is determined. The second module implements an unsupervised raking CNN network to recognise the emotion and intensity estimation using ordinal evolution in the sequence data.

All of the stated approaches fail to adequately model the human mental capacity of predicting emotion with their respective intensity. The methods either estimate emotion intensity without emotion recognition or recognise emotion and its intensity separately. None of the methods carries out both tasks simultaneously. Multilabel learning is the recent trending approach to FER. This approach emerges from the public opinion that facial expression image contains a mixture of emotion, and only in a rare occasion is pure emotion displayed in face (Plutchik, 2001; Yannakakis, Cowie & Busso, 2017).

Facial expression challenges influenced FER system’s performance, and the efforts in the field tend towards how the challenges could be reduced to bearable minimal. In the FER research community, diverse approaches have been implemented to enhance or optimise CNN networks to mitigate FER challenges. Some of the CNN optimisation approaches focus on improving the network’s discriminating power through modification of loss function to reduce intraclass variance and increase interclass variance. Loss function guides the optimisation function in the direction to follow, and it states how close or far is the model prediction to the ground truth. The traditional loss function for multiclass tasks is softmax loss (Liu et al., 2016; Wang et al., 2018). The challenge identified with softmax loss is that while penalising the misclassified samples, it repels different classes to cluster apart, which is a challenge in FER, the introduction of center loss function aid to alleviate softmax loss challenge in the sense that it was able to cater for intraclass variation but fails to consider interclass variation appropriately. As discussed in Cai et al. (2018), island loss is capable of increasing network discriminating power by increasing interclass variation and reducing intraclass variation, which is the main challenge in FER tasks. The experiment conducted in Cai et al. (2018) island loss function shows a better performance than either softmax loss or centered loss function. Likewise, Li & Deng (2019) in their effort to implement a robust FER with high discriminating power, form a tuplet cluster loss function, which is a hybrid of a tuplet (N+1) loss function and cluster loss function. The (N+M) tuplet cluster loss described an N-negative and M-positive sample in the CNN framework’s minibatch. The formed tuplet cluster is combined with softmax loss as a joint optimisation technique to explore identity label and expression label information potentials thoroughly.

Other modification of CNN networks is found in Alenazy & Alqahtani (2020), Ozcan & Basturk (2020), Wu, Wang & Wang (2019) and Zatarain Cabada et al. (2020). Ozcan & Basturk (2020) improved FER system performance with transfer learning and hyperparameter tuning. Alenazy & Alqahtani (2020) present a semi-supervised deep belief network for FER and employed gravitation search algorithm for network parameter optimisation. Wu, Wang & Wang (2019) optimise CNN network for FER classification by converting the output layer tensor of the network into a multidimensional matrix-vector *via* matrix transformation to enlarge the eigenvalues such that the system might have lower loss rate. Zatarain Cabada et al. (2020) proposed a genetic algorithm optimisation technique for CNN hyperparameter tuning for FER. The main goal of the genetic algorithm is to achieve the best solution from the hyperparameter population evolution.

This work is presenting an enhanced ML-CNN model for emotion recognition and ordinal intensity estimation. The proposed multilabel deep learning model can learn the hierarchical structure in FER datasets during the training of the network and predicts the emotion and the ordinal intensity in the expression face concurrently. Transfer learning optimisation technique is used as a trade-off for the insufficient data population for the appropriate ML-CNN model learning. The entropy loss function is fortified with island loss function to minimise the intraclass and interclass challenges. Detail description of our model is presented in the next section.

## Multilabel convolution neural network model (ml-cnn) description

Deep learning models are traditionally employed in solving either a binary class or multi-class problems, where an instance of a population is only restricted to a group of class. In such a multitask challenge a single output is generated. Very few studies considered deep learning for multilabel tasks. Liu et al. (2016) practically established this fact that facial expression in the real world is more of mixture or compound of emotion. Their work verified this while using the Expectation-Maximization (EM) algorithm to automate the manual annotation of Real-world Affective Faces (RAF) database. Their approach shows that expression face contains more than one emotional state in different intensity level.

ML-CNN is a deep learning model we consider for classifying expression images into the emotional states and the associated degree of intensity. ML-CNN model combines multilabel problem transformation techniques with CNN algorithm as a deep learning technique for the multilabel classification task. Details of this model are considered in the following subsections.

### Problem transformation

**Figure 2:**
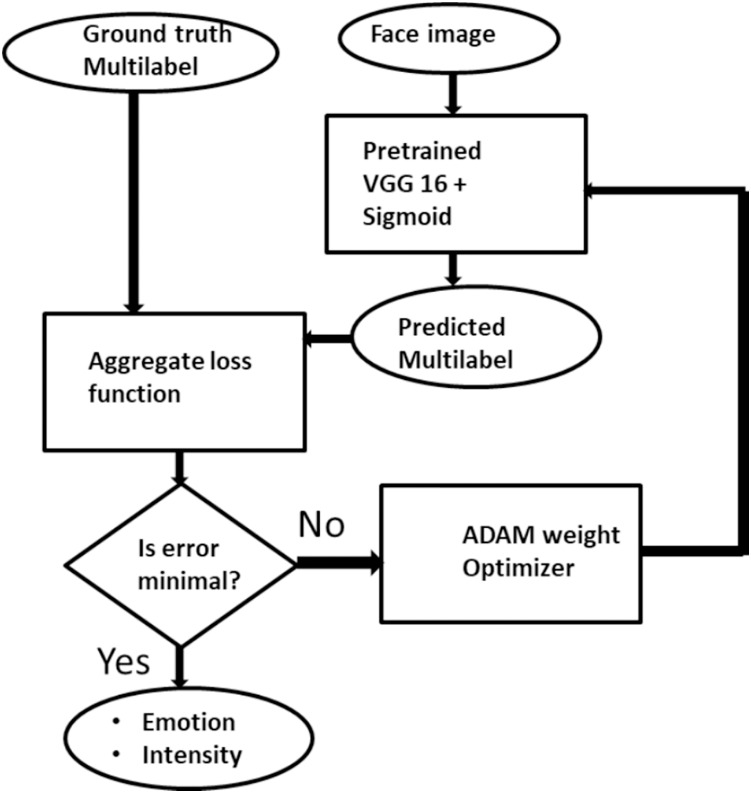
The description of Multilabel CNN model for facial expression recognition and intensity estimation.

Here, we formally present facial expression and intensity estimation task as a multilabel problem. Generally, assume *X* = *R*^*m*^ represents set of training samples with m dimensional feature vectors, a sample *x* ∈ *X* associated with a label *y* ∈ *Y* is given as *E* = {*x*_*i*_; *y*_*i*_} such that ${y_i} \subseteq k$ where *k* = {*y*_*i*_: *j* = ,…*p*} is the set of *p* possible labels. In the context of facial expression recognition and intensity estimation, a special multilabel scenario is defined. An expression image is associated strictly with emotion information *y*_*i*_∈ *Y* and intensity information *z*_*i*_ ∈ *Z*. Formally, given an Expression image *E* = {*x*_*i*_, *y*_*i*_ × *z*_*i*_} where *y*_*i*_ × *z*_*i*_ ∈ *Y* × *Z* such that *k*_1_ = $\{ y\} _{i = 1}^P$ for all possible *p* ∈ *Y* and ${k_2} = \{ z\} _{i = 1}^q$ for all possible *q* ∈ *Z*. The challenge in this multilabel task is to generate a supervised classifier *C* which is capable of taken an unseen expression image *E* and simultaneously predict its correct emotion state and its intensity. That is, given *E* = (*x*_*i*_,) Then *C*(*E*) → *Y* × *Z*, which is the accurate emotion and intensity associated with the image. This transformation is achieved with binary relevance extension transformation technique as proposed by Luaces et al. (2012) with a slight modification that limits label independence. Binary relevance also aids in adopting deep learning into the multilabel environment. Figure 2 gives the pictorial description of the proposed ML-CNN model.

### Convolution neural network multilabel adaptation

**Figure 3:**
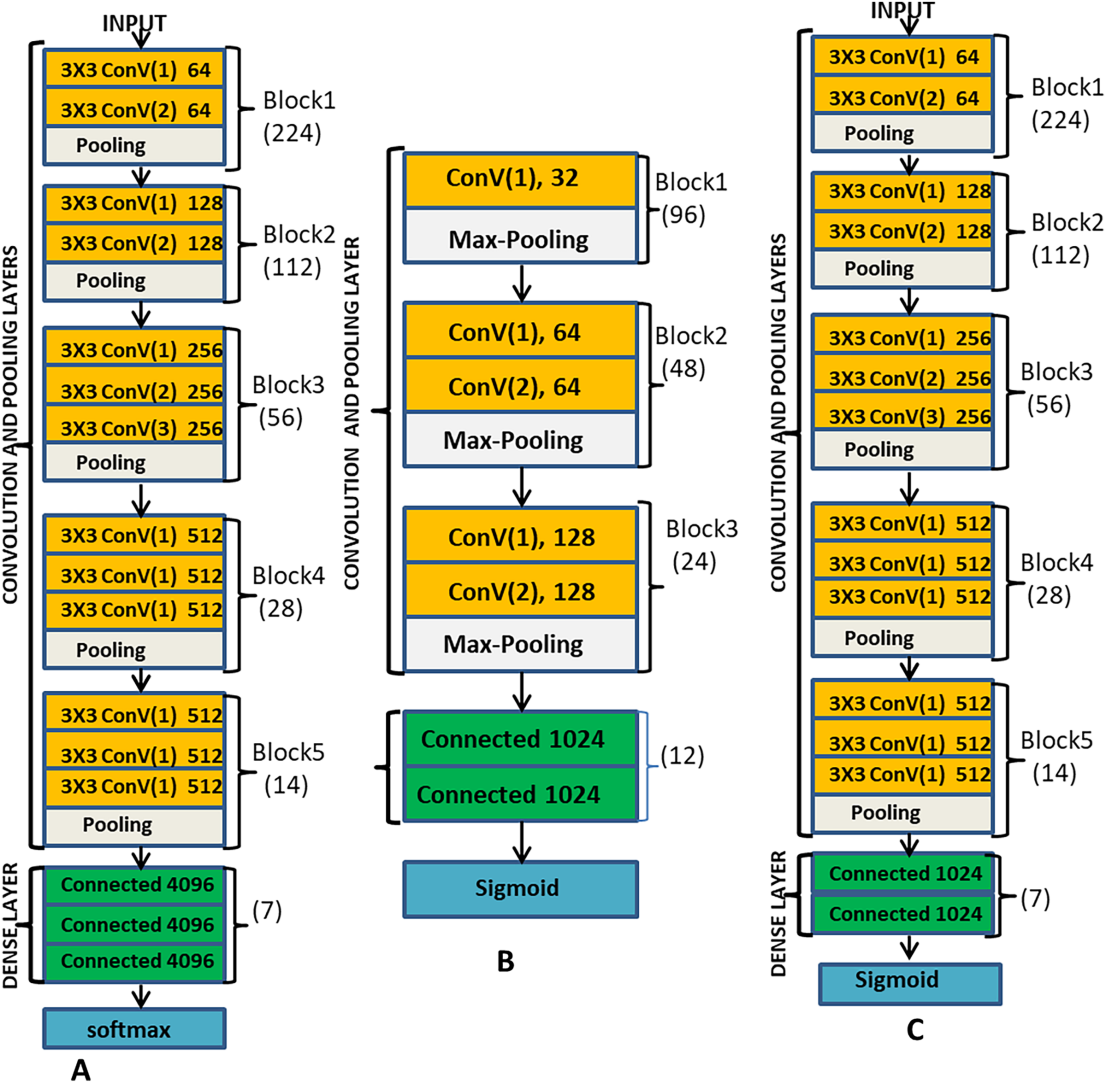
(A) Description of VGG-16 model; (B) the proposed ML-CNN modeland; (C) the VGGML-CNN model, which the optimised version of ML-CNN.

The main components of CNN include the convolution layers, the pooling layers, the fully connected layers and the output layer. ML-CNN model is designed similarly with VGG network but with a fewer number of blocks. Figure 3B illustrates the arrangement of all the components of ML-CNN.

Convolution Layer: Convolution layer deals with the extraction of representative features from the expression image; it performs convolution operation on the input image to preserve the spatial relationship between pixels. With convolution operation, local dependencies of the input image are learned. Convolution operation involves convoluting input data with a filter to give a corresponding output which size is determined by some parameters like depth, stride and zero paddings. Convolution layer also employs activation function, which is continuous and differentiable for learning a non-linear transformation of the input data and enhances the network to access a rich hypothesis space from deep representation. This work employs 3 × 3 kernel, ReLu activation function, zero-padding one stride and batch normalisation at each convolution layer. There are five convolution blocks in this model, and the first convolution layer convolutes the input image with the kernel to produce 32 feature maps, a non-linear activation function ReLu is applied to learn the non-linearity features, sparsity control and also to prevent gradient vanishing which is likely to occur during back-propagation. For the stability of each layer, we also used batch normalisation and 0.5 dropout. All these operations took place at each of the convolution layers, except that, different filters are generated at other convolution layers. At the second and third convolution layer, 64 feature maps are produced, and at the fifth and sixth layers, 128 feature maps are produced.

Pooling Layer: this is a sub-sampling layer of the network where the down-sampling operation takes place. Its goal is to reduce feature maps’ dimension and ensure the preservation of the most useful feature. Pooling operation reduces the computation complexity by reducing the number of training parameters, reducing distortion, and rotation, translation and scaling sensitivity. This system employs max-pooling methods. In the max-pooling feature, maps are convoluted with a 2 × 2 kernel to return the maximum value from each region covered by the kernel. This network contains three pooling layers, and the first pooling layer is positioned after the first convolution layer, the second and the third pooling layers are after the third and the fifth convolution layer respectively as shown in Fig. 3B.

Fully Connected layer: This layer behaves like feed-forward network, the output of the last pooling layer is flattened, that is the 2-dimensional matrix is unrolled into a vector. This is because fully connected layer takes a one-dimensional matrix as input, the flattened function converts the height, the width and the feature maps into a series of feature vectors (*h* × *w* × *f*). This layer also used ReLu activation function and 0.25 dropout.

Classifier: The last layer of ML-CNN, which is the output layer is a sigmoid classifier, and the sigmoid activation function can generate an independent probability for each of the classes and thus suitable for the multilabel classification task.

Loss Function: Loss function guides the optimisation function in the direction to follow, and it states how close or far is the model prediction to the ground truth. Here, Adaptive Moment (ADAM) optimisation function is considered with learning rate of 0.001. ML-CNN is a multilabel model which implements sigmoid activation function at the output layer, the most appropriate loss function for ML-CNN is Binary Cross-Entropy (BCE) loss. BCE combines the functionality of sigmoid activation function and cross-entropy function in the sense that the loss computes for a class has no effect on the loss computes for other classes, and also form a binary classifier between each of the classes and background class, which is not a member of the classes in consideration. With BCE, loss calculated for each class is independent on the other classes, BCE is formally expressed in (1).

Deep learning networks performance has been enhanced in literature by the modification or introduction of some loss functions like: triplet loss (Chen et al., 2020; Dong & Shen, 2018; Vijay Kumar, Carneiro & Reid, 2016; Cheng et al., 2016), center loss (Wen et al., 2016), and Island loss (Cai et al., 2018). As discussed in Cai et al. (2018), island loss is capable of increasing network discriminating power by increasing interclass variation and reducing intraclass variation. This is the main challenge in FER tasks especially in our model where intraclass variation is large among the representative image samples because each of the classes contains different subjects, and small interclass variation is observed between classes because subjects are the same for all classes. An experiment conducted by Cai et al. (2018) showed that island loss function is better in performance than softmax loss function or with center loss function.

(1) $$BCE({s_i}) = - \sum\limits_{i = 1}^{C = 2} {t_i}log({s_i})$$

where *s*_*i*_ is the model score and *t*_*i*_ is the ground truth for each class *i* ∈ *C*.

This work is adapting island loss to enhance the choice of discriminating features in the ML-CNN model. Island loss is an improvement over the center loss with the tendency to minimise or avoid overlapping of different clusters, thus increasing interclass variations. Just like the presentation in Cai et al. (2018), We follow similar steps and positioned island loss function after the fully connected layer. The island loss function is formally expressed in (2).

(2) $${{\rm {\cal L}}_{IL}} = {{\rm {\cal L}}_C} + {\lambda _1}\sum\limits_{{c_j} \in N} \sum\limits_{{c_k} \in N,{c_k} \ne {c_j}} \left(\displaystyle{{{c_k}.{c_j}} \over {||{c_k}{{||}_2}||{c_j}{{||}_2}}} + 1\right)$$

${{\rm {\cal L}}_C}$ is the center loss expressed in (3), expression label set is represented with N, both c_*k*_ and *c*_*j*_ indicate the two center terms with *L*_2_ norm ||*c_k_*||_2_ and ||*c_j_*||_2_ that penalise the expression of different samples and the similarity of expression samples from the center respectively. *λ*_1_ is to balance *c*_*k*_ and *c*_*j*_

(3) $${{\rm {\cal L}}_c} = \displaystyle{1 \over 2}\sum\limits_{i = 1}^m ||{x_i} - {c_{yi}}{||^2}$$

ML-CNN model implements BCE loss function at the final layer, then the entire loss function of ML-CNN is provided in (4).

(4) $${\rm {\cal L}} = {{\rm {\cal L}}_{{\rm BCE}}} + \lambda {{\rm {\cal L}}_{IL}}$$

where ${{\rm {\cal L}}_{BCE}}$ is Binary Cross Entropy loss, and *λ* is a hyper-parameter for balancing the two losses. The implementation procedure of ML-CNN is detail in Algorithm 1.

**Algorithm 1:** ML-CNN algorithm.

**Input:** Training Data X = {*xi*,(*yi* × *zi*)}
**Output:** Network layer parameter W, L_IL_, L_BCD_
1 Given: minibatch n, learning rate *α*, *μ* and hyperparameter *λ* and *λ*_1_
2 Initialization: {t, W, *θ*, *c*_*j*_}
3 t = 1
4 while(t != T) {compute the aggregate loss Lagg = L_BCE_ + *λ*_LIL_
5 update L_BCE_
6 ${\gamma ^{t + 1}} = {\gamma ^t} - \mu (\partial L_{BCE}^t)/(\partial {\gamma ^t})$
7 update *L*_IL_
8 *cj*^*t*^ ^+ 1^ − *α* Δ*cj*^*t*^
9 update backpropagation error
10 $\partial {L^t}/\partial x_i^t = \partial L_{BCE}^t/\partial x_i^t + \lambda (\partial L_{IL}^t/\partial x_i^t)$
11 Update network layer parameter
12 ${W^{t + 1}} = {W^t} - \mu \partial {L^t}/\partial {W^t} = {W^t} - \mu (\partial {L^t}/\partial x_i^t)(\partial x_i^t/\partial {W^t})$
13 *t* = *t* + 1}

### Transfer learning

Transfer Learning could be thought of as a way of preventing re-inventing the wheels in computer vision, in the sense that knowledge of a particular deep model could be transferred or reuse more especially in a similar environment or for a related task. Transfer learning mechanism improvises for data challenges in computer vision, and it is considered as one of the deep learning optimisation techniques for addressing overfitting effect in the field. Adapting the knowledge or weight of pre-trained standard deep network into a related task or challenge is the main concept of transfer learning. Few of the standard deep pre-trained networks include: VGG Network (VGGNET) (Simonyan & Zisserman, 2015), Residual Network (ResNet) (He et al., 2016), Inception_w (Szegedy et al., 2017; Szegedy et al., 2016), Google Network (GoogLENet) (Szegedy et al., 2015) and the likes.

ML-CNN design is a similitude of VGG; we consider VGG-16 as a pre-trained network for our model. The pre-trained network is adapted as a feature extractor for our ML-CNN. The fully connected layers and the ML-CNN classifier control the learning and the interpretation of the extracted features on the datasets and preserve both the multilabel learning and independent classification. Figure 3 is the pictorial description of the VGGML-CNN model. Figure 3A is the description of VGG-16 model, Fig. 3B is the proposed ML-CNN model, and Fig. 3C is the VGGML-CNN model, the optimised version of ML-CNN.

## Experiment

### Preprocessing

Deep learning is known for its autonomous feature extraction capability. Despite, observations show that there is an improvement in networks performance when data is preprocessed. Data preprocessing advantages to deep learning include minimization of computational costs (computational time and computational resources) and availability of proper representative features that is noise-free. In this work, we find it appropriate to employ some preprocessing techniques to aid our model sensitivity in the automatic feature extraction phase. This section carried out two essential data preprocessing techniques: face localization (face detection) and face augmentation.

#### Face localization

Face detection is about locating the region of a face from an image, sequence of images or video. Face detection algorithms are often involved in virtually most face related research in computer vision such as face recognition, Age estimation from face, image-based gender recognition and facial expression recognition. All these tasks consider face detection as one of the main steps in their preprocessing stage. In this study, we consider the face detection algorithm proposed by Viola & Jones (2001). The only modification to this algorithm is the implementation of an integral graph for eigenvalues computation as in Zhang, Jolfaei & Alazab (2019) which aid the computation speed, we use the method to compute Haar-like feature *via* integral graph as shown in Eq. (5). In the process, relevant features of Haar-like are carefully selected and later integrated into a robust classifier with the aid of the AdaBoost algorithm.

(5) $$G(x,y)I(x,y) = \sum\limits_{{x}^{\prime} \le x,{y}^{\prime}} i({x}^{\prime},{y}^{\prime})$$

where I(.) is the integral image and i(.) is the real image.

#### Augmentation

Augmentation is one of the policies employed in computer vision to alleviate data limitation challenge. Data augmentation is mostly used in deep learning where there is a need for extensive data size for deep learning model to learn the representative feature appropriately from the data sample when training the network. Data augmentation could be implemented on the fly or off the fly. This work implements off the fly techniques using the augmentor module in python3 for data balancing among the classes.

### Experimental databases

(1) Binghamton University 3D Facial Expression (BU-3DFE): BU-3DFE (Yin et al., 2006) is a controlled static dataset that captured real-world challenges. It consists of 2,00 data collected form 100 subjects. Each of the subjects produced four images for each of the six basic emotion classes (Anger, Disgust, Fear, Happy, Sadness, Surprise) with their respective intensity annotated with ordinal metrics. BU-3DFE is the only FER dataset that considers ordinal intensity annotation in the database to the best of our knowledge.

(2) Cohn Kanade Extention (CK+): CK+ (Lucey et al., 2010) is a sequence dataset and well-annotated into seven basic expression classes (Anger, Disgust, Contempt, Fear, Happy, Sadness and Surprise). It is made up of 327 sequence data collected from 118 subjects. A subject produced an emotion sequence for each of the seven basic emotions starting from the neutral face (offset) to the onset and apex. CK+ is a popular dataset for facial intensity estimation, and for this study, the data is organised following the flow of changes in the sequence to have an ordinal label in substitute for the onset, offset and apex. The sequence of expression for each subject is categorised into four ordinal intensities {Low, Normal, High, and V High} according to the observed changes. This implies that each emotion will have four sub-classes tagged with the emotion and each of the ordinal intensities. For instance, a subject with an anger expression sequence would be grouped into AngerLow, AngerNormal, AngerHigh and AngerVery High. The arrangement makes CK conform to the ordinal intensity arrangement in BU-3DFE datasets.

### Experiment procedures

This section evaluates the proposed ML-CNN model and the comparative study of its performance with the existing multilabel models. BU-3DFE and CK+ data are the set of databases employed for the experiments. After pre-processing, each of the raw data was scaled to a uniform size of 96 × 96. The pixel values were divided by 255 to ensure data scale normalisation. The datasets are partitioned into the training set (70%), the validation set (20%), and the remaining 10% is the testing set. The experiment was conducted using OpenCV, Scikit-learn, Keras with TensorFlow 2.0 backend. All the required software were installed on High-Performance Computing (HPC) hardware resources at the Center for High-Performance Computing (CHPC).

Evaluation of ML-CNN model begins with training procedure. The model was first trained on the training data division and evaluated on the validating data severally with some modifications to the model parameters to minimise the model’s over-fitting. Adam optimiser with initial learning rate of 0.001 is used. Initially, we consider the model’s performance evaluation on the BU-3DFE data with a data size of 2,400. We also extend the experiment to observed the system performance when the training data is augmented. The augmentation is implemented offline to ensure data balance among the classes. Here, we evaluate the system performance on both BU-3DFE and CK+ datasets. We also observed the transfer learning optimisation technique on the model by fine-tuning the model with a pre-trained VGG-16 CNN network model. We employ accuracy and the loss (binary cross entropy and island loss) metrics for the model performance evaluation on the testing data in each of the described experiment.

The other phase of our experiment is a comparative study of the ML-CNN and four different other multi-label algorithms: RAKELD (Distinct Random k-Label sets) (Tsoumakas, Katakis & Vlahavas, 2011), classifier chain (CC) (Read et al., 2009), MLkNN (Multilabel k Nearest Neighbour) (Zhang & Zhou, 2007) and MLARAM (Benites & Sapozhnikova, 2015). To avoid bias, the algorithms were implemented in the same environment and executed on similar datasets with fair consideration by using multilabel performance evaluation metrics. Gaussian Naive Bayes is the based classifier in RAKELD, the base classifier for CC is the random forest, while kNN is used as the base classifier for MLkNN nearest neighbour k is set to 10, and smoothing parameter is 1. The multilabel metrics used for our models’ comparative studies with other models include average precision, hamming loss, coverage error, and ranking loss. The following section contains a brief discussion of each of the listed multilabel metrics.

### Evaluation metrics

**Hamming Loss**: is computed using the XOR operator as the loss between the predicted and actual labels. The Hamming loss is defined in Eq. (6).

(6) $$H = \displaystyle{1 \over {|N|.|L|}}\sum\limits_{i = 1}^{|N|} \sum\limits_{j = 1}^{|J|} XOR({y_{i,j}},{\hat y_{i,j}})$$

**Ranking Loss**: computes the average of the incorrectly ordered labels. The smaller the Ranking loss, the better the performance of the model. The Ranking loss is defined in Eq. (7).

(7) $$Ran{k_{loss}}(y,\hat f) = \displaystyle{1 \over N}\sum\limits_{1 = 0}^{N - 1} ||{y_i}{||_0}\displaystyle{1 \over {k - ||{y_i}{{||}_0}}}|Z|$$

where *k* is the number of labels and *Z* is (m,n): $\hat {\rm f}$_i,m_ *≥* $\hat {\rm f}$_i,n_, y_i,m_ = 1, y_i,n_ = 0

**Average Precision**: is the number of higher-ranked labels that are true for each ground-truth label. The higher the Average precision value, the better the performance of the model. The ranking average precision is defined in Eq. (8).

(8) $$LRAP = \displaystyle{1 \over N}\sum\limits_{i = 0}^{N - 1} {\displaystyle{1 \over {||y||}}_0}\sum\limits_{j:{y_{i,j}} = 1} \displaystyle{{|{L_{i,j}}|} \over {Ri,j}}$$

where L_i,j_ = K: y_i,k_=1, $\hat {\rm f}$_i,k_ *≥* $\hat {\rm f}$_i,j_ and |.| is the cardinality of the set.

**Coverage Error**: computes the number of labels required to included all the correct labels in the final prediction. The smaller the value, the better the model performance of the model. The Coverage error is defined in Eq. (9).

(9) $$Coverage(y,\hat f) = \displaystyle{1 \over N}\sum\limits_{i = 0}^{N - 1} maxran{k_{i,j}}$$

where rank_i,j_ is—{k: $\hat {\rm f}$_i,k_ *≥* f_i,k_—}

In addition to the comparative studies, we visually observed the model prediction output and compared the degree of intensity predicted for each expression with the truth label.

## Experimental results and discussion

The experiments’ results are summarised in the figures and the tables below. The experiments first observe ML-CNN model’s performance and the optimisation technique adopted on both the BU-3DFE and CK+ datasets. We use accuracy and the loss function as the model evaluation metrics. Observations showed that ML-CNN based model provides a training accuracy of 95% and validation accuracy of 88.56%, training loss and validation loss of 0.142 and 0.3534, respectively. Augmentation of training data improves ML-CNN performance with about 2% increase in training accuracy and almost 4% increase in validation accuracy. The result obtained by fine-tuning ML-CNN with VGG network improves the model performance with validation accuracy close to 8%. The summary of these results is presented in Table 2. Table 2 shows that our model outperforms some existing methods on facial expression recognition and intensity estimation. Although the methods in consideration either recognised expression before the intensity estimation or model the intensity estimation before expression recognition, which is quite different from our model that recognises expression and intensity concurrently.

**Table 2:** The tabular presentation of ML-CNN and VGGML-CNN performance evaluation Using accuracy and aggregate loss on BU-3DFE and CK+ datasets, and their comparison with some existing methods. In the table, metric with ↑ indicates that the higher the metric value the better the model performance, and metric with ↓ indicates that the lower or smaller the value of the metric the better the model performance.

ML-Models	Database	Accuracy ↑	Aggregate loss ↓
ML-CNN	BU-3DFE	88.56	0.3534
	AUG_BU-3DFE	92.84	0.1841
	CK+	93.24	0.2513
VGGML-CNN	Bu-3DFE	94.18	0.1723
	AUG_BU-3DFE	98.01	0.1411
	CK+	97.16	0.1842
Kamarol et al. (2017)	CK+	82.4	NA
Walecki et al. (2015)	CK+	94.5	NA
Quan, Qian & Ren (2014)	CK+	88.3	NA

**Figure 4:**
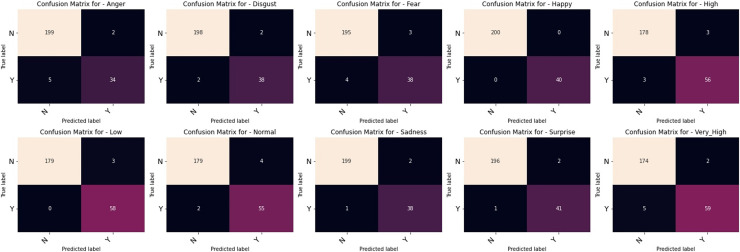
Multilabel confusion matrix of the VGGML-CNN on Bu-3DFE.

**Figure 5:**
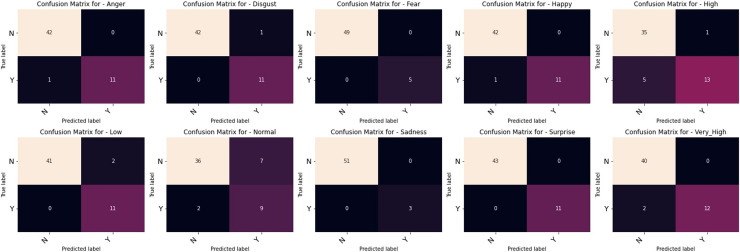
Multilabel confusion matrix of the VGGML-CNN on CK+.

The outcomes of our comparative studies of ML-CNN models with some other multilabel algorithms are presented in Tables 3–5. It is evident from the tables that our proposed multilabel model shows a better performance than the multilabel algorithms considered. Table 3 indicates that ML-CNN and VGGML-CNN give outstanding performances over RAKELD, CC, MLkNN and MLARAM when predicting emotion and the degree of intensity BU-3DFE. Observation from Table 4 clearly showed that RAKELD, CC, MLkNN and MLARAM degrade in performance on Augmented BU-3DFE data, unlike ML-CNN VGGML-CNN that showed significant improvement in their performance under similar conditions. Table 5 also shows that both VGGML-CNN and ML-CNN outperformed other multilabel algorithms. Furthermore, Tables 6 and 7 contain the detail predictions of each expression and intensity on the test samples of the datasets. Figures 4 and 5 presents the multilabel confusion matrix for VGGML-CNN performance on both the BU-3DFE and CK+ respectively. VGGML-CNN performance is compared with some of the recent models of FER using CK+ and BU-3DFE datasets. VGGML-CNN shows outstanding performance on BU-3DFE, and a good result on CK+, detail of the comparative study is presented in Tables 8 and 9.

**Table 3:** The result of the comparative studies of multilabel models’ performances on BU-3DFE dataset is presented as follows. Metric with ↑ indicates the higher the metric value, the better the model performance, and metric with ↓ indicates the lower or smaller the value of the metric the better the model’s performance.

ML-Models	Hamming loss ↓	Ranking loss ↓	Average precision ↑	Coverage ↓
RAKELD	0.4126	0.6859	0.2274	4.8137
CC	0.1807	0.8393	0.3107	4.8094
MLkNN	0.1931	0.8917	0.2634	4.9486
MLARAM	0.3045	0.6552	0.3180	3.1970
ML-CNN	0.1273	0.2867	0.5803	2.5620
VGGML-CNN	0.0890	0.1647	0.7093	1.9091

**Table 4:** The comparative studies of multilabel models’ performances on augmented BU-3DFE dataset are presented as follows. Metric with ↑ indicates the higher the metric value, the better the model performance, and metric with ↓ indicates the lower or smaller the value of the metric the better the model’s performance.

ML-Model	Hamming loss ↓	Ranking loss ↓	Average precision ↑	Coverage ↓
RAKELD	0.3858	0.7223	0.2241	4.0453
CC	0.1825	0.8948	0.2812	4.7270
MLkNN	0.1929	0.9025	0.2573	4.9623
MLARAM	0.3169	0.6963	0.3280	2.9315
ML-CNN	0.1124	0.2278	0.7216	2.2397
VGGML-CNN	0.0628	0.1561	0.8637	1.3140

**Table 5:** The result of the comparative studies of multilabel models’ performances on CK+ dataset is presented as follows. Metric with ↑ indicates the higher the metric value, the better the model performance, and metric with ↓ indicates the lower or smaller the value of the metric the better the model’s performance.

ML-Model	Hamming loss ↓	Ranking loss ↓	Average precision ↑	Coverage ↓
RAKELD	0.3904	0.6637	0.2370	4.4435
CC	0.1489	0.6842	0.4234	4.7339
MLkNN	0.1839	0.8345	0.2965	4.7930
MLARAM	0.1951	0.4636	0.4144	3.0748
ML-CNN	0.1487	0.4161	0.5926	2.8120
VGGML-CNN	0.1393	0.3897	0.6002	1.4359

**Table 6:** Emotion and intensity degree predictions on BU-3DFE test samples.

EMotion and ordinal intensity	Accuracy %
Anger	97.0
Disgust	98.3
Fear	97.0
Happy	100
Sadness	98.7
Surprise	98.7
Low	98.7
Normal	97.5
High	97.5
Very High	97.0

**Table 7:** Emotion and intensity degree prediction on CK+ test samples.

Emotion and ordinal intensity	Accuracy %
Anger	98.1
Disgust	98.1
Fear	100
Happy	98.1
Sadness	100
Surprise	100
Low	96.2
Normal	83.3
High	87.0
Very High	96.3

**Table 8:** Comparison result of VGGML-CNN with some recent models on CK+.

Model	Accuracy %	No of classes	Target
Cai et al. (2018) (IL-CNN)	94.35	7	Expression only
Li & Deng (2019) (DLP-CNN)	95.78	7	Expression only
Alenazy & Alqahtani (2020) (DBN-GSA)	98	7	Expression only
Xu et al. (2020) (CCNN)	91.50	6	Expression and intensity
Chen et al. (2020) LDL-ALSG	93.08	7	Expression distribution
ML-CNN	93.24	6	Expression and intensity
VGGML-CNN	97.16	6	Expression and intensity

**Table 9:** Comparison result of VGGML-CNN with some recent models on BU-3DFE.

Model	Accuracy %	No of classes	Target
Fernandez et al. (2019) (FERAtt)	85.15	7	Expression only
Shao & Qian (2020) (MVFE-LightNet + Residual Convolution)	88.70	6	Expression only
Bao, Zhao & Chen (2020) (CNM)	80.63	6	Expression only
VGGML-CNN	98.01	6	Expression and intensity

### Equity and model bias

Although BU-3DFE is static data, also regarded as data-in-the-wild, the data comprises of subjects of different ages, ethnicity, races, and genders. Other factors that possibly challenge FER recognition are considered in the collection of the data. The result of the model on BU-3DFE shows that human variation factors have limited effect on the model.

## Conclusions

This work proposed a new approach to FER and intensity estimation. The multilabel convolution neural network (ML-CNN) method employed problem transformation technique and used CNN as the binary classifier to predict emotion and its corresponding intensity estimation using ordinal metrics. For system robustness and accuracy reliability, we used transfer learning optimisation as a trade-off for the small data population and overfitting prevention. We modified the loss function by introducing island loss function to enhance the model sensitivity to intraclass variation minimisation and interclass variation maximisation. Our proposed model accurately predicts the emotional state with the corresponding degree of intensity concurrently. From the comparative study of ML-CNN with other multilabel algorithms, ML-CNN shows significant performance advantage, more especially with both augmented data and the model optimisation. Despite the excellent performance, the ML-CNN model still finds it difficult to generalise to unseen data outside the scope of the databases used. We suspect infiltration of person specificity, that is, personal identity into the training time model, as the possible reason. The drawback should be considered in the future work for a robust ML-CNN model that will generalise well with unseen data. In addition, future work should also consider using some spontaneous data-in-the-wild that have no hierarchical intensity organization, such as FER2013 and FER+, and dynamic FER data that would support the real-life application of the model.

## Supplemental Information

10.7717/peerj-cs.736/supp-1 Supplemental Information 1 Python code for the project.

## Additional Information and Declarations

### Data Availability

The following information was supplied regarding data availability: The data we used are available at: - BU-3DFE: http://www.cs.binghamton.edu/~lijun/Research/3DFE/3DFE_Analysis.html - CK+: CK+ is available upon approved request: https://www.jeffcohn.net/resources/ Our Python code is available as a Supplemental File.
